# The Government and Vaccination

**Published:** 1899-02-18

**Authors:** 


### The Hospital

A Journal of

The Medical Sciences and Hospital Administration.

Vol. XXV.—No. 647.] Edited by Sir Henry Burdett, K.C.B., and Solomon C. Smith, M.D., M.R.C.P. [Feb. 18, 1899.

### The Government and Vaccination.

It has recently been stated in several newspapers that Mr. Boulnois, the Conservative member for East Marylebone, intended to introduce into the House of Commons a Bill for the repeal of the "conscientious objector" clause of the Vaccination Act of last Session; and it was suggested that he was influenced in this resolution by the large number of medical men in his constituency. Mr. Boulnois, if he entertained the intention attributed to him, has not been fortunate in the ballot by which the place of any legislative proposal emanating from a private member is determined; and in the meanwhile a question asked by Mr. Bartley has enabled the President of the Local Government Board to explain the position of the Government in relation to the question. Mr. Chaplin told the House that the influence of the conscience clause has been much less important than is commonly supposed, and he showed that the decline of vaccination, when studied by the light of the statistics in the possession of the Department, is seen to have been progressive for many years. Going back to 1885, so as to include all children at present under fourteen years of age, he said that the number remaining unvaccinated had steadily increased from year to year, and had risen from 136,000 in 1885 to 370,000 in 1898. More than twelve and a half millions of births were registered during the period included between the two dates; and of these the children left unvaccinated amounted to more than three millions and a quarter (3,235,000). Up to January 1st the total number of certificates of conscientious objection amounted to rather more than a quarter of a million (239,000), so that in round numbers the new Act is only answerable for having legalised about one-seventh of the neglect that has been suffered to exist. In these circumstances, and bearing in mind that the Act only came into full operation six weeks ago, Mr. Chaplin declared that he had not sufficient information to enable him to form any confident opinion as to its working, or to justify any proposal for its modification during the present session. The reply, of course, will determine the attitude of the Government with regard to any attempts at legislation proceeding from private members. Mr. Chaplin added that the demands upon the Local Government Board for glycerinated lymph have become so great as to render necessary a large increase in the staff engaged in its production and distribution; and he derived from this fact some ground for the hope that vaccination is actually on the increase, as compared with recent years.

As far as all reasonable members of the community are concerned, it is impossible to doubt that such an increase might be legitimately looked for as a natural result of the report of the Royal Commission, and of the various channels through which the main features of that report have been rendered accessible to the public. It has long been necessary to admit that in some rare cases vaccination has been attended by certain dangers, as from erysipelas and from the inoculation of accidental by-products; and these admissions have probably influenced the minds of many persons who have had no means of estimating the infinitesimal smallness of the risks concerned. Now that those of the latter class have been entirely obviated, and those of the former have been shown to depend upon disregard of cleanliness, the absolutely safe and unobjectionable character of the operation, as well as its practical efficacy, stand out clearly from the maze of controversy in which the whole question has been involved. The professional anti-vaccinators, of course, will continue their exhortations, at all events, for the present; but, it may fairly be hoped, with a continually diminishing measure of success. Like Demetrius and his fellow silversmiths at Ephesus, "by this craft they have their living," often their mere physical living, and in other cases the sole support of their reputations as publicists or philanthropists. In proportion to their activity, the owners of property, the directors of insurance offices, the managers of schools, and the employers of labour may properly exercise increased stringency with regard to the vaccination of tenants, policy-holders, pupils, and servants, so that, after all, the consequences of the conscience clause may ultimately prove less disastrous than was feared. We cannot help deriving another kind of consolation from the figures which Mr. Chaplin laid before the House. Fielding, in a play performed one hundred and sixty years ago, makes a spendthrift enlarge upon his prospect of succeeding to the estate of his uncle, who "hath but five children, and but one of them hath had the small-pox." The argument was then sufficiently valid, and may at any rate serve to illustrate the risks from which the present generation has escaped by aid of the protection given by vaccination, a protection, however, which would seem to be rapidly fading away if it be true that there are now about three millions of unvaccinated children and young people in England.

